# Understanding the Risks and Benefits of Implementing AI-Enabled Remote Patient Monitoring Systems for Disease Management

**DOI:** 10.3390/ijerph22111734

**Published:** 2025-11-17

**Authors:** Junaid Nabi, Richard Staynings, Javaid Iqbal Sofi, Henry H. Willis

**Affiliations:** 1 RAND School of Public Policy, Santa Monica, CA 90401, USA; 2 The Aspen Institute, Washington, DC 20037, USA; 3 Cylera, Inc., New York, NY 10005, USA; 4 The University of Denver, Denver, CO 80210, USA; 5 Virginia Polytechnic Institute and State University, Blacksburg, VA 24061, USA; javaidiq@vt.edu; 6 RAND Corporation, Santa Monica, CA 90401, USA

**Keywords:** algorithmic accountability, machine learning systems, patient safety, policy analysis, regulatory frameworks, AI-enabled remote patient monitoring, risk analysis, risk mitigation, cybersecurity, technology adoption

## Abstract

Effectively managing risk is essential for fostering innovation in healthcare, especially with advancements like artificial intelligence (AI) and machine learning (ML). These technologies aim to enhance accessibility, efficiency, and equity in healthcare delivery. To assess the practical utility of AI-enabled remote patient monitoring (RPM) devices, it is crucial to identify and evaluate associated risks while distinguishing between acceptable risk, which society tolerates, and optimal risk, which balances risk reduction costs with benefits. This paper outlines how policymakers should adopt the framework of optimal risk to ensure patient safety while maximizing the advantages of these technologies.

## 1. Introduction

Remote patient monitoring (RPM) is of growing importance across healthcare delivery as nurses retire from the industry and are increasingly difficult to replace especially in developed countries. Combined with nursing shortages, hospital efficiency drivers are pushing hard for higher levels of automation in the orchestration of patient monitoring and basic telemetry data collection. AI-enabled Remote Patient Monitoring (RPM) shows great promise in helping to meet these hospital objectives, while current adoption has thus far been perceived very positively. This paper discusses the drivers for AI-enabled RPM and examines the risks to AI-based technology in a healthcare delivery environment already suffering from inadequate cybersecurity and resiliency, while being the target of increasingly devastating cyber-attacks.

The objective of this paper is to analyze the significant benefits and complex risks associated with AI-enabled RPM systems and propose a practical “optimal risk” framework designed for policymakers and healthcare leaders to guide safe and effective implementation of these technologies.

## 2. Realistic Motivations for the Development of AI-Enabled RPM

Understanding the necessity for developing AI-enabled Remote Patient Monitoring (RPM) technologies requires examining the fundamental pressures driving healthcare innovation. The widespread adoption of these platforms represents a direct response to mounting challenges within modern healthcare systems that demand immediate attention.

The growing burden of chronic diseases—particularly cardiovascular, diabetic, and hypertensive conditions—places unprecedented economic strain on healthcare infrastructure worldwide. Ninety percent of the nation’s $4.9 trillion in annual health care expenditures are attributed to people with chronic and mental health conditions [1]. The estimated cost of chronic disease is expected to reach $47 trillion worldwide by 2030 [2]. Chronic heart failure alone affects 2.5% of American adults, with costs predicted to reach $70 billion by 2030 [3]. This exponential growth in patient populations occurs alongside limited capacity for in-person care within existing healthcare systems, creating an unsustainable gap between patient needs and available resources.

## 3. The Business Case for AI-Enabled Patient Monitoring

### 3.1. Efficiency Gains

AI-enabled Remote patient monitoring (RPM) has greatly helped drive efficiency, automation and the quality of care provided to patients. This includes a growing number of elderly, terminal, and in-home patients with chronic or acute illnesses supported by remote clinical care teams [4]. AI-enabled RPM has facilitated the early hospital release of post-operative patients to those exercising their right to die in their own homes and everyone in between [5]. It has also revolutionized in-hospital care by attaching IoT devices and sensors to patients to report vital signs while fitting outpatient rooms with AI-enabled cameras that monitor the physical condition of patients including behavioral analysis, movement and fall detection.

### 3.2. Speedier Intervention

AI-enabled RPM has successfully combined advanced medical telemetry from pulse oximeters, blood glucose meters, blood pressure monitors, and many other devices used to monitor vitals, with logic to automate the time-consuming job of monitoring patients while instantly alerting staff when intervention is needed [6]. This has improved the efficiency of nursing units allowing a greater number of patients to be monitored by a single nursing station while improving intervention speed thanks to instantaneous and accurate alerts. Like all new technologies however, AI enablement and automation can introduce risks, especially agentic systems that are relied upon to automatically and accurately alert clinicians.

### 3.3. Cost Savings

Before analyzing the risks presented by AI-enabled RPM, it would be helpful to analyze the benefits of this modality. A balanced assessment is required to understand the true impact of technological innovations [7]. AI-enabled RPM presents immense opportunities in early detection and timely interventions, as these systems are capable of detecting early signs of change in patient condition and disease exacerbation. This is achieved by the continuous assessments of large volumes of data collected by the devices in close proximity to the patient, as well as the information that the patients themselves report [8]. This enables appropriate timely clinical interventions that lead to improved patient outcomes, which, in the long run, also enable cost savings.

### 3.4. Improved Diagnosis and Outcomes

Another unique benefit of employing AI-enabled RPM modalities is the ability of clinicians to make accurate diagnoses and adjust management plans for patients, which is a result of these tools using large quantities of real time data [9]. This is possible because AI-enabled RPM can be integrated with the electronic health records at the clinician’s hospital. These integrations also enable clinicians to develop individualized treatment plans for patients (Personalized Medicine), as the AI-enabled RPM devices can analyze the vital signs and medical history of the patient [10]. These benefits further enhance the advantages of minimizing the risk of hospital-acquired infections and preventing unnecessary hospitalizations due to minor changes in treatment plans. Lastly, these devices enable real time monitoring of a patient’s routine health status. For example, they can accurately detect and record vital metrics such as heart rate, blood pressure, oxygen levels, and other important indicators that are often overlooked in the management of the patient’s clinical condition.

## 4. Evaluating Healthcare Risk in the Context of New Technology

Risk is an inevitable consequence of innovation. Effectively managing risk is, therefore, a necessary condition of managing innovation—and ensuring greater benefit for a larger number of people.

### 4.1. Powerful New Tools

The healthcare industry has witnessed a massive influx of advanced digital technologies in the last few years, from continuous biomarker sensors (for diabetes patients) to robotic surgery for cancer patients [11]. Now with the introduction of artificial intelligence (AI)—machine learning (ML) and deep learning (DL) technologies, there is a renewed hope to improve accessibility, efficiency, and equity in the healthcare delivery system [12]. Integrating these technologies into the emerging area of AI-enabled Remote Patient Monitoring platforms presents a unique opportunity to leverage these powerful digital tools to enable continuous surveillance of patients outside the confines of the traditional hospital system—presenting economic and clinical benefits but also challenging traditional notions of risks and benefits of providing clinical care [13].

### 4.2. Balancing Risk and Reward

While there is an active discussion of these technologies within the context of clinical use in the realm of published research, there is limited understanding of how the introduction of these tools can lead to data security problems. This is a consequence of increased and sustained digitization of healthcare services, which offers significant convenience for patients, while simultaneously exposing a greater volume of sensitive data to potential cyberattacks. This heightened vulnerability increases the risk of security breaches, which can result in devastating financial and reputational losses for healthcare organizations and erode patient trust, as was recently seen in the case of Change Healthcare data breach in 2024 [14]. Consequently, mitigating these risks to achieve fewer cybersecurity breaches is a critical challenge for the evolving healthcare landscape.

### 4.3. Watershed Moments in Cyber Security Risk

The significance of the Change Healthcare breach, which was a watershed moment in decades of escalating healthcare cyber-attacks, was in Change Healthcare’s status as a critical third party and single point of failure for thousands of healthcare providers, pharmacies, and other life sciences organizations. The Change Healthcare outage directly and negatively impacted hundreds of US providers while inconveniencing thousands more with a single ransomware attack. A cyber-attack that ultimately impacted close to 195 million patients whose records were compromised, and which denied, or delayed many urgently needed medical procedures and pharmaceutical prescriptions [15]. It also prevented many providers and pharmacies from gaining payer pre-approvals for treatment and prescriptions, placing undue risk upon those delivery partners. The impact of this attack is ongoing with many pharmacies and medical providers yet to be fully reimbursed for drugs and procedures over a year later, while loans and emergency payments from UGH are still being disputed through the courts [16].

Finally, the attack, and the outage the attack caused, may result in increases in patient morbidity and mortality as a result of denied or delayed medical interventions, as well as the closure of some providers for unreimbursed procedures and pharmacies for unpaid prescriptions [17]. The extensive operational and financial disruptions that the Change Healthcare attack resulted in lasted for 6 months or longer and is being blamed for the closure of multiple rural hospitals and clinics, along with numerous independent pharmacies.

### 4.4. Single Points of Failure

The dangers of single critical points of failure across the infrastructure of US healthcare seems obvious but is being largely ignored by government regulators until very recently [18]. Over the past decade there has been a massive consolidation and concentration of healthcare payers, providers, and pharmacy benefit managers (PBMs) under, chiefly, three healthcare umbrella corporations. There has also been a vertical integration of payers, providers and PBMs, blurring the division of functions and consolidating massive levels of protected health information (PHI) and other data under one roof making each a huge target for perpetrators [19].

One of these consolidations is Change Healthcare, which was bought in 2022, despite the Justice Department suing to block the acquisition by United Healthcare Group (UHG), owner of United Health (payer) and Optum (provider) [20]. It was under Optum’s management that the Change Healthcare application was successfully cyber-attacked. The danger with ongoing consolidation across the US healthcare industry is that new companies and new highly innovative technologies could be quickly consumed by one of these corporations and become an equally vulnerable single point of failure as Change Healthcare proved to be [21]. These concerns include AI-enabled RPM, where economic drivers could quickly propel a single vendor provider of these services to a similar criticality.

### 4.5. Too Many Eggs in One Basket

The entire US healthcare industry is based upon an unhealthy concentration of a few key providers of critical technologies and services. This includes an effective duopoly of electronic medical record (EMR) vendors—EPIC and Oracle/Cerner, and an oligopoly of healthcare payers and PBMs. In early 2025 the Oracle Cloud supporting its EMR was breached. The hackers claimed to have breached over 6 million medical records, but the full impact of the attack is still under investigation [22].

### 4.6. Lack of Resiliency

Internally, each healthcare provider through years of cost constraints, has developed an equally unhealthy reliance upon single vendors for its technologies and in many cases medical and other vital supplies [23]. Parallel suppliers are an unknown luxury across this industry. When the supply chain breaks as it did during the COVID-19 Pandemic, or when critical vendor services and systems are impacted by a cyber-attack, then healthcare delivery organizations also suffer and are unable to meet patient and community needs [24]. The industry is simply unprepared to quickly switch out one vendor for another in times of difficulty, as the Change Healthcare attack and many other such attacks have proven. Nor does surplus capacity exist for alternate vendors providing comparable services when a major player suffers difficulty or a sustained outage.

Unlike other industries, healthcare has failed to invest in an n + 1, or n + 2, high resiliency application mix, or in highly resilient network and infrastructure architectures. The result is that single points of failure are ubiquitous, and the right attack could easily take down the entire system nationwide [25].

### 4.7. Critical National Infrastructure and Hybrid Attacks

Rising geopolitical tensions makes healthcare a prime target [26]. This is not a risk unique to US healthcare providers. The UK has also suffered from devastating cyber-attacks, one against Synnovis in June 2024, which took down pathology services for two London NHS Trusts killing one patient and injuring 14 others [27], and another in January 2023 against the UK Royal Mail, two weeks after the UK government agreed to provide additional assistance to Ukraine [28]. Indeed, rising threats of hybrid warfare, cybercrime, and cyberterrorism should all play into the risk equation for the acceptance of new medical technologies [29].

### 4.8. Acceptance and Public Trust

Innovation must be balanced with assurances of privacy, security, and compliance. It must also be resilient and able to withstand rising cyber-attacks. Acceptance of technologies that result from innovation may be contingent upon public trust in those assurances [30]. Indeed, the widescale adoption of new higher-risk technologies will be dependent upon greater assurances of cybersecurity and safety than are demanded from current medical technologies given a growing climate of attacks against healthcare providers and an increasingly risk-averse general public [31]. There is a constant ongoing tension between risks and benefits in the domain of rapidly evolving technologies, as the pace of advancements in these innovations frequently outstrip society’s capacity to assess, evaluate, and eventually manage the associated risks. Consequently, it is necessary for clinicians—even those who may not be involved in the technical development of AI-enabled RPM platforms—to broadly understand the risks involved in these platforms and develop an informed understanding of how these emerging risks may be mitigated [32]. Equally, user feedback is an essential component to redesigning and updating these platforms, which can lead to safer and improved outcomes for patients and ease of use for clinicians [33].

## 5. Evaluating ‘Acceptable’ and ‘Optimal’ Risks to Artificial Intelligence

To fully conceptualize the practical utility of AI-enabled RPM devices and to conduct a comprehensive risk assessment, it will also be necessary to identify and measure risks associated with this evolving technology. This is important because policymakers and innovators must be able to differentiate between *acceptable* and *optimal* risk [34].

Acceptable risk refers to the level of risk that society is willing to tolerate, while optimal risk aims to find a balance between the costs of risk reduction and the benefits it brings. This involves minimizing negative outcomes and maximizing positive ones, based on defined criteria like costs, legal and compliance requirements, and patient outcomes.

Recent policy changes at the FDA, require device manufacturers to build security into their products from the design phase and maintain it throughout the product’s lifecycle. They also require the publication of a software bill of materials (SBoM) and regular risk disclosures for new devices. The rules do not apply retroactively to the millions of legacy medical devices. This is all part of FDA’s ‘Refusal to Accept’ policy changes for 510(k) approval that were mirrored by the International Medical Device Regulators Forum (IMDRF) [35,36]. While this is still considered a shared responsibility with healthcare delivery organizations (HDOs) which must be vigilant about patching connected medical devices and implementing their own network security, including network segmentation and network access control (NAC) [37], the onus for maintaining optimal and acceptable risk has clearly shifted towards manufacturers under the governance of the FDA and other national regulatory bodies. This will include devices employed for AI enabled RPM.

### 5.1. CIA Triad and Security Risk & Compliance

Protecting the Confidentiality, Integrity, and Availability (CIA) of health data and systems is the basis of both effective risk analysis and meeting healthcare regulation [38]. While regulation in most countries is myopically focused upon the protection of confidentiality and privacy of personal health information (PHI) [39], there is a growing alarm to protect the other two sides of the CIA triad. Of particular concern is availability of medical systems that provide access to clinical data, as ransomware attacks attempt to deny such access by extortion. Additionally, there is a risk of data integrity compromise, and that false or deliberately misleading information could be introduced to the patient record through a cybersecurity infiltration, or that automated systems fail to recognize and alert clinical staff to changes in a patient’s condition [40].

### 5.2. Protecting the Integrity of Artificial Intelligence

As medical AI systems grow in adoption, so do their security algorithms, and the clinical data used for training is becoming an increasing concern. A growing proliferation of false, fake, and misleading data is entering both academic and mainstream bodies of knowledge. Conference papers, academic articles, and scientific books have all had to be withdrawn when it was later discovered that they were based upon false or fabricated data and premise [41,42]. The number of fraudulent scientific papers is rapidly increasing and is now thought to be doubling every year and a half [43]. Chinese paper mills have been especially prolific at churning out research papers written entirely by large language models (LLMs) and lacking original research or accuracy [44]. While LLMs often draw the wrong conclusions from their data sources, so too do medical AI applications when data is tainted or corrupted [45]. *Adversarial machine learning* and *data poisoning* are just two of many weaknesses that could have real world impacts on AI-based health information technology systems, and consequently upon patient safety and clinical outcomes [46]. These AI risks extend even to simple monitoring and patient telemetry systems.

With healthcare delivery becoming increasingly digitized, cybersecurity breaches are a constant threat, especially because health data is much more sensitive and permanent than personal finance data. A victim’s credit card number can be easily swapped out and changed, whereas their genome is unique and unalterable [31] (p. 144). This will require implementation of robust encryption, data anonymization, and “need to know” data access protocols in healthcare settings. Above all, maintaining security of health data is critical to foster patient trust [47].

### 5.3. Remote Risks to AI-Enabled RPM Devices

Any medical device or RPM system that operates outside of the confines of a hospital or other healthcare delivery facility faces additional security concerns as it will usually not be protected by an enterprise corporate firewall, nor full-time professional security and networking teams. Patient telemetry systems operating in the home will usually be connected back to a hospital or nurse monitoring center via the home’s consumer internet service provider. Most of the time, this will be using an inexpensive consumer router-firewall that is often insecure, unpatched, misconfigured, and woefully out of date on its operating firmware. Even the tunneling of data across a virtual private network (VPN) runs the risk of PHI data being intercepted or that the remote network could be compromised and used as foothold by attackers [48].

‘Right to Die at Home’ laws in certain jurisdictions, notably, the UK, Australia, and New Zealand, allow terminal patients the right to die in the familiar surroundings of their own homes surrounded by photographs, family, and pets. Healthcare providers are required under these laws to facilitate necessary RPM and nurse call systems in addition to any remotely managed pain medication devices. However, this has caused many security problems for providers to both protect the patient’s PHI, and to protect the integrity of core provider networks from remote network attacks [49].

Indeed, the acceptance of new technologies especially those that are based upon or employ AI, is increasingly being considered through the lens of cybersecurity, privacy, and benefits versus risk, [50]. Many highly innovative ideas are never brought to market because of concerns of privacy, security, or regulation so ensuring security really is a prerequisite to adoption [31] (pp. 148–149).

### 5.4. The Importance of Training Data

AI has an insatiable need for data; data for training, data for ensuring large sample sizes to remove bias, and data for driving improvements to natural language processing. Small data samples have proven ineffective for ML training. Small sample sizes also raise concerns that algorithms could be reverse engineered or that the identity of individual patients discovered. For these reasons, AI requires huge amounts of data for training to ensure accuracy. These requirements make it difficult to share raw medical data with researchers unless assurances can be made to protect healthcare regulated data in accordance with regulations [31] (p. 145).

A related severe risk associated with these systems is the potential to amplify threats to critical infrastructure [51]. AI systems that are integrated into essential services, such as healthcare delivery, can become targets for attacks. Adversaries may manipulate AI models through poisoning or by using adversarial inputs, which could disrupt these vital services [51]. This is evidenced by worryingly common ransomware attacks on healthcare facilities in recent years—leading to disturbances in patient care delivery, reputational damage, and severe financial losses. 

Furthermore, AI-enabled technologies are completely dependent on the quality of data used, and as decision makers think about protecting the data from breaches, there must also be a concerted effort to maintain high quality data that feeds the algorithms [51]. Suboptimal data can lead to inaccurate outcomes from the devices and eventually lead to improper clinical care. To address this risk requires using diverse and durable training datasets and establishing a dedicated schedule for continuous validation to maintain reliability of the devices and accuracy of the clinical endpoints being captured.

### 5.5. Algorithmic Bias

Another major risk category is the potential of these devices to create healthcare inequities or lead to algorithmic bias, especially for vulnerable communities [52]. A “digital divide” already exists in the healthcare sector between those who can readily access healthcare through technology platforms and those who are unable to do so for a variety of social and economic reasons. AI-enabled RPM devices also pose the risk of exacerbating this digital divide and expanding healthcare disparities. This is because AI algorithms are developed using specific datasets, and when these datasets reflect inherent biases within the healthcare system, the algorithms may perpetuate and even intensify these biases [53]. As an example, if an AI algorithm that is intended for the detection of heart failure is primarily trained on data from a specific demographic or racial group, it may exhibit reduced accuracy in diagnosing heart failure in other patient populations. This situation can lead to disparities in access to timely and appropriate care, particularly impacting marginalized and underrepresented communities. As a result of concerns around algorithmic bias, marginalized and underrepresented communities are often less trusting and less willing to share their medical data with data scientists to improve bias. These dynamics present an ethical dilemma and an operational challenge to address these concerns [54].

While the risks of AI-enabled RPM—from cybersecurity to algorithmic bias are significant, it is in the crucible of real-world application that their true potential and challenges come into focus. Nowhere is this more apparent than in the Intensive Care Unit (ICU), a data-rich, high-stakes environment that serves as a critical proving ground for these emerging technologies. To move beyond theoretical discussion, this next section examines concrete examples of how advanced AI-enabled RPM systems are being deployed and governed in critical care, providing an essential practical foundation before we consider a broader policy framework.

## 6. Mitigating and Optimizing Risks in AI-Enabled RPM

Effectively managing the novel risks posed by AI requires a deliberate shift away from static risk models toward more adaptive governance frameworks. The World Health Organization’s Ethics and Governance of Artificial Intelligence for Health guidance emphasizes that AI technologies must put ethics and human rights at the heart of their design, deployment, and use, providing six consensus principles to ensure AI works for the public benefit [55]. Similarly, the European Union’s AI Act establishes a risk-based legal framework, classifying AI-enabled medical devices—including remote patient monitoring systems—as high-risk AI systems subject to strict requirements for safety, transparency, data governance, and human oversight [56]. These frameworks emphasize that ethical considerations and risk management must be integral to AI system design and deployment, not afterthoughts.

### 6.1. Adaptive Frameworks

To illustrate this framework, we examined several representative cases from recent literature and regulatory reports, including Vent.io, CLEWICU, and COMPOSER**,** which were selected to demonstrate the diversity of risks across different AI-enabled RPM applications. This is particularly true for “Software as a Medical Device” (SaMD), where an AI model’s performance can evolve—or degrade—as it encounters new real-world data. In response, regulatory bodies like the U.S. Food and Drug Administration (FDA) have begun to champion concepts like the Predetermined Change Control Plan (PCCP). This approach is a practical application of the ‘optimal risk’ framework described earlier, as it seeks to continuously balance the benefits of an evolving AI model against the risks of performance degradation. A PCCP is essentially a living document that outlines how an AI/ML model is allowed to be updated post-deployment—specifying what can be modified, the methodology for implementing changes, and how the impact will be assessed—all while maintaining the device’s safety and effectiveness.

The clinical utility of this approach is clearly demonstrated in a study on “Vent.io,” a machine learning model designed to predict the need for mechanical ventilation [57]. Their work included a robust PCCP which stipulated that if the model’s predictive accuracy (its AUC) fell below a set threshold, it would automatically trigger a retraining process. While the model performed well in its initial deployment, its performance dropped significantly when tested against an external dataset from a different hospital system. This performance drop when encountering a new patient population directly illustrates the risk of algorithmic bias and the ‘digital divide’ highlighting why adaptive governance is essential. The model’s PCCP was activated, and the subsequent retraining successfully restored the model’s accuracy. By ensuring continuous validation against real-world data, the PCCP addresses the critical need for data quality and reliability, while its transparent structure helps mitigate the ‘black box’ problem, fostering the clinical trust necessary for adoption [58].

### 6.2. Reinforcement Learning & Adversarial Attacks

Big concerns exist around the issues of adversarial attacks in the field of reinforcement learning where an AI is set in continuous learning mode. Many such AI models have been easily corrupted through deliberate adversarial inputs in the form of adding tiny perturbations to inputs which can lead a model to give wrong results [59]. Attacks include label poisoning, dataset poisoning, white box and black box adversarial attacks.

### 6.3. The Intelligent Intensive Care Unit

The integration of AI in the ICU extends beyond predictive algorithms to physical devices and comprehensive sensing platforms. Systems like the “Intelligent Intensive Care Unit” utilize sensor-equipped carts to conduct real-time visual and environmental assessments [60], while other platforms employ on-device video analysis to passively monitor patients for signs of delirium or unsupervised movement [61]. These tools augment clinical staff by detecting subtle patterns that might be missed during standard periodic checks.

AI and RPM integration shows particular promise in time-sensitive conditions. Take sepsis, for example. Deep-learning models like COMPOSER are now being deployed to analyze real-time data streams from ICU monitors, predicting the onset of sepsis hours in advance. Clinical evidence suggests such systems are associated not only with improved compliance with sepsis treatment bundles but also with reduced in-hospital mortality [62,63].

In a similar vein, AI models can now predict the likelihood of Acute Kidney Injury (AKI) before serum creatinine levels rise significantly, allowing for preventative interventions [64]. Likewise, models that forecast the need for mechanical ventilation up to 24 hours in advance, such as Vent.io, provide clinicians with a critical window to optimize care and allocate resources efficiently [57]. Beyond specific organ systems, FDA-cleared platforms like CLEWICU analyze integrated data patterns to provide warnings of general hemodynamic instability up to eight hours in advance [65]. Other models specialize in predicting ICU delirium, giving care teams the foresight needed to implement proactive mitigation strategies [66].

### 6.4. Adaptive Risk Management

What these examples collectively demonstrate is that the conversation around AI-enabled RPM is maturing rapidly. In the high acuity setting of the ICU, we are witnessing a powerful convergence of predictive analytics and adaptive risk management. The success of these systems is not based on the algorithm alone, but on its thoughtful integration within a clinical workflow and a governance structure like the PCCP that accounts for the inevitability of change. This practical reality—that maximizing benefit requires actively and continuously managing risk—provides the essential context for the broader policy discussion that must follow. Of course, the successful deployment of these platforms is contingent not only on clinical integration and adaptive governance but also on addressing the profound cybersecurity challenges inherent in remote systems.

Additional compounding risks from the implementation of AI technology within RPM devices are described in Table 1 below. A supplemental comparative analysis also illustrates how these benefits and risks compare with traditional RPM devices to create a comprehensive understanding of the issue. As decision makers assess the risks from these innovative devices, they also should be mindful of the availability heuristic—where people judge risks based on the ease with which examples come to mind, leading to overestimation of highly publicized events (cybersecurity breaches in healthcare settings) and underestimation of less dramatic risks, such as complications from chronic diseases [67].

## 7. Limitations

The authors acknowledge certain limitations in this analysis. This paper presents a conceptual framework and policy perspective; it is not based on new empirical data collection or analysis. The case examples are included for illustrative purposes to highlight key risk categories and are not intended as a systematic literature review. Future research should focus on empirical studies to validate the proposed optimal risk framework quantitatively and assess its practical application in real-world clinical settings.

## 8. Conclusions

As has been the case for numerous technological innovations before, the advent of AI-enabled RPM platforms has presented clinicians and healthcare administrators with a remarkable opportunity to dramatically improve patient outcomes, reduce costs, and alleviate the burden on healthcare delivery systems—now, the next step is to find a balance between the benefits and risks. These leaders and practitioners can use the discussion in this paper to formulate policies that can help them to minimize the risks of these technologies and maximize the benefits.

## Figures and Tables

**Table 1:** A comparative analysis of risks and benefits between AI-enabled remote patient monitoring (RPM) devices and traditional RPM devices.

Feature	AI-Enabled RPM	Traditional RPM	Potential Benefits of AI vs. Traditional RPM	Potential Risks of AI vs. Traditional RPM
Data Analysis	Clinical pattern recognition; anomaly detection; accurate risk prediction	Manual data system: time consuming; error prone	Early identification; opportunity for proactive interventions	Exacerbation of health inequities from “machine bias”
Personalization	Individualized clinical insights and treatment recommendation/options	Treatment options are generic, and not for individual	Enhanced patient engagement and treatment compliance	“Black box” nature can impede development of trust
Efficiency	Data analysis can be automated; decreased documentation burden for clinicians	Manual review and analysis of data; increased documentation burden for clinicians	Increased time for patient–doctor interactions	Technology reliance can hamper clinical judgement
Patient Engagement	Automatic reminders and nudges can improve patient compliance to management plans	Patient engagement takes immense resources/time	Patients play an active role in managing their health	Technology problems can lead to delayed care or undertreatment of medical condition
Data Security	High risk of cybersecurity breaches as more patient data is digitized and vulnerable	Fewer cybersecurity breaches	Digitized healthcare delivery is more convenient for digitally savvy patients	Cybersecurity breaches can lead to massive financial/trust losses

## Data Availability

No new data were created or analyzed in this study.
